# The Role of Matrix Metalloproteinase Polymorphisms in Ischemic Stroke

**DOI:** 10.3390/ijms17081323

**Published:** 2016-08-12

**Authors:** Jason J. Chang, Ansley Stanfill, Tayebeh Pourmotabbed

**Affiliations:** 1 Department of Neurology, University of Tennessee Health Science Center, Memphis, TN 38104, USA; 2 Department of Nursing and Department of Genetics, Genomics and Informatics, University of Tennessee Health Science Center, Memphis, TN 38104, USA; astanfi4@uthsc.edu; 3 Department of Microbiology, Immunology and Biochemistry, University of Tennessee Health Science Center, Memphis, TN 38104, USA; tpourmot@uthsc.edu

**Keywords:** ischemic stroke, matrix metalloproteinase, polymorphism, incidence, epidemiology

## Abstract

Stroke remains the fifth leading cause of mortality in the United States with an annual rate of over 128,000 deaths per year. Differences in incidence, pathogenesis, and clinical outcome have long been noted when comparing ischemic stroke among different ethnicities. The observation that racial disparities exist in clinical outcomes after stroke has resulted in genetic studies focusing on specific polymorphisms. Some studies have focused on matrix metalloproteinases (MMPs). MMPs are a ubiquitous group of proteins with extensive roles that include extracellular matrix remodeling and blood-brain barrier disruption. MMPs play an important role in ischemic stroke pathophysiology and clinical outcome. This review will evaluate the evidence for associations between polymorphisms in *MMP-1*, *2*, *3*, *9*, and *12* with ischemic stroke incidence, pathophysiology, and clinical outcome. The role of polymorphisms in MMP genes may influence the presentation of ischemic stroke and be influenced by racial and ethnic background. However, contradictory evidence for the role of *MMP* polymorphisms does exist in the literature, and further studies will be necessary to consolidate our understanding of these multi-faceted proteins.

## 1. Introduction

Cerebrovascular disease (CVD) is characterized by brain ischemia that results in focal, acute neurological deficits and remains the fifth leading cause of death in the United States [1]. Over eighty percent of CVD consist of ischemic stroke (IS), defined by focal blood vessel occlusion. The main mechanisms of IS are thrombosis, embolization, and lacunar infarction that result in oxygen loss and ATPase dysfunction. The resulting cerebral ischemia initiates several pathological events including blood-brain barrier (BBB) disruption, vasogenic edema, secondary hemorrhagic transformation, and neuronal death.

The principal risk factors for IS incidence remains the metabolic syndrome, characterized by the co-incidence of hypertension, diabetes, obesity, high triglycerides, and low high-density lipoprotein [2,3]. More recently, IS incidence has also been found to be associated with environmental exposures such as alcohol, smoking, diet, and exercise [4,5].

However, not all individuals exposed to similar environmental factors suffer equally from IS. Differences in incidence, pathogenesis, and clinical outcome have long been noted when comparing IS among patients with different racial and ethnic backgrounds. Epidemiological studies highlight significant racial disparities with blacks having significantly higher stroke incidence compared to whites [6], younger age of incidence [7], and poorer functional outcomes [8].

Still, clinical, environmental, and demographic risk factors do not fully explain disparities in IS disease progression [9]. As with many other diseases, IS results from the interaction between an individual’s genetic makeup and environmental exposures [10]. For instance, high-density lipoprotein cholesterol and smoking in blacks, systolic blood pressure in Asians, and age in both Hispanics and blacks resulted in different atherosclerotic disease progression compared to whites [11]. Asians and Hispanics were found to have slower progression of carotid intima-media thickness, and blacks had less carotid plaque formation [12]. Finally, one study showed that blood pressure significantly correlated with stroke incidence in South Asian males, but not in European males [13]. Improved understanding has suggested a role for genetics in IS susceptibility [14].

Significant research has been carried out to establish the relationship between functional variants of different genes and IS risk [15,16,17,18,19,20,21]. One such potential genetic risk factor may be *matrix metalloproteinase* (*MMP*) polymorphisms [22]. The role of *MMP* polymorphisms in gene transcription and their associations with various diseases has been evaluated. Roles for specific *MMP* polymorphisms have been found in cancer incidence [23], coronary artery disease [24], and glaucoma [25]. Not surprisingly, burgeoning research in *MMP* polymorphisms for various types of populations have been conducted in IS as well. This review will evaluate *MMP* polymorphisms and provide some insight into their roles in IS incidence and clinical outcome.

## 2. Background on Matrix Metalloproteinases

MMPs are members of a unique family of zinc-binding endopeptidases that are secreted as catalytically latent species and processed to their activated forms in vivo by other proteases, the tissue plasminogen activator-plasmin system, or post-translational modifications [26,27,28]. At least twenty-five different MMPs have been identified [29]. Members of this protease family have been divided into five subclasses based on structural similarity and substrate specificity that include the following: collagenases (MMP-1, MMP-8, and MMP-13) [30,31,32], gelatinases (MMP-2 and MMP-9) [33,34,35], stromelysins (MMP-3 and MMP-10) [36,37], metalloelastases [38,39], membrane-type MMPs (MT-MMP, MMP-14, MMP 15, MMP-16, MMP-17, MMP-24, and MMP-25) [40,41,42,43,44,45], and others (MMP-7, MMP-11, MMP-12, MMP-19, MMP-20 and MMP-23) [46,47].

As ubiquitous proteins, MMPs have extensive roles that include epithelial repair against pathogens in innate immunity [48,49], control of chemokine activity (including CCL-2, 7, 8, and 13) [50], and the activation of inflammatory cytokines (interferon-β, vascular endothelial growth factor, epidermal growth factors, fibroblast growth factors, and transforming growth factor-β1) [51]. However, MMPs also play a significant role in normal and pathological conditions that involve ECM degradation and remodeling [52].

MMPs have also been shown to be involved in the complex pathophysiology of IS. Mechanisms of MMP in IS include atherosclerotic plaque maturation, plaque degradation and rupture [53], development of subclinical periventricular white matter disease (i.e., leukoaraiosis) [54], and hemorrhagic transformation (HT) particularly after thrombolysis [55]. While MMP-8 [56] and MMP-10 [57] have been related to atherosclerosis and brain ischemic, respectively, MMP-1, 2, 3, 9, and 12 have been most identified in the pathophysiology of IS. We will discuss each of these in turn.

## 3. Matrix Metalloproteinase-1

MMP-1 is a 53 kDa protein that plays an active role in the degradation of interstitial collagen types I, II, and III, and major structural components of the fibrous plaque, which forms the mechanism behind atherosclerotic stroke. The active-site zinc works with a hinge region and carboxyl terminal domain to unwind the triple-helical collagen structure [58]. Although implicated in neuronal cell death [59], MMP-1 has also been closely linked to advanced atherosclerotic plaques and has been postulated to exacerbate atherosclerosis by degrading plaques, which leads to a cycle of plaque expansion and rupture [53].

### Clinical Role of MMP-1 Polymorphisms

A single-guanine (1G to 2G) polymorphism located at the *MMP-1* promoter region -1607 1G/2G has been identified. The 2G-allele of this promoter has been noted to increase transcriptional activity by creating an E26 transcription factor binding site [60]. But Chehaibi et al. evaluated the role of this 1G vs. 2G allele in IS incidence in Tunisian patients and did not find any association between the -1607 promoter gene polymorphism and IS incidence [61]. But this failed association might be due to the similarities in genotype frequency between patients with carotid artery atherosclerosis and controls (*p* = 0.085). However, *MMP-1* 2G/2G homozygotes and *MMP-3* polymorphisms were shown to synergistically lead to an increase in carotid atherosclerosis (odds ratio (OR) 3.31, *p* = 0.004) [62].

To date, no clinical study has evaluated the role of *MMP-1* polymorphisms in clinical outcome of IS, and their role in IS incidence remains undetermined.

## 4. Matrix Metalloproteinase-2

MMP-2 (gelatinase-A) is a 72 kDa protein that degrades collagen IV, fibronectin, laminin, aggrecan, gelatin, elastin and non-matrix substrates, latent TGFβ9, monocyte chemoattractant protein-3, fibroblast growth factor receptor 1, big endothelin-1, and plasminogen. MMP-2 has several roles in IS. It disrupts the BBB and leads to HT [63]. It is also extensively associated with leukoaraiosis. This has been shown in animal models utilizing *MMP-2* knockout mice and the corresponding inhibitors that significantly decreased chronic white matter disease [64]. Pathological studies demonstrated that MMP-2 levels are higher in patients with vascular dementia [65] and lacunar strokes [66]. Yet, MMP-2 serum levels immediately after acute IS are not markedly elevated [66,67]. However, the activity of MMP-2 increases in the late phase of IS [66,67,68], suggesting that it plays a neuroprotective role within the ischemic core.

### Clinical Role of MMP-2 Polymorphisms

Five *MMP-2* polymorphisms have been found to be associated with the development of lacunar strokes [69]. However, only a few *MMP-2* genetic studies demonstrate its role in atherosclerotic disease. Price et al. identified a -1306 (C vs. T allele) polymorphism in the promoter region of *MMP-2*. This C to T transition disrupted the promoter, leading to lower *MMP-2* expression and enzymatic activity [70].

One mechanism potentially linking MMP-2 with increased IS incidence is its association with leukoaraiosis. Zhang et al. evaluated the -1306 C/T promoter of *MMP-2* in a Chinese population and found the CC genotype resulted in higher transcriptional activity and independently predicted leukoaraiosis (*p* = 0.027) [71]. However, Nie et al. evaluated this same promoter in a Chinese population and did not find an association between the CC genotype and IS incidence [72]. Instead, they found the -735 C-allele (C vs. T allele) in the *MMP-2* promoter region was associated with a greater incidence of IS (OR 1.516, 95% confidence interval (CI) 1.185–1.940, *p* = 0.001) in their Chinese population. The C-allele of the -735 promoter is associated with significantly higher *MMP-2* transcriptional activity [72]. High MMP-2 production and activity may be one risk factor for IS incidence.

Unlike other MMPs, the presence of elevated MMP-2 after IS in the acute phase has been associated with improved clinical outcome [66]. Manso et al. found significant associations between two single-nucleotide polymorphisms (SNP) rs2241145 and rs1992116 and clinical outcome after Bonferroni correction. For rs2241145 (G vs. C allele), the G-allele was significantly associated with good outcome after stroke (OR 1.66, 95% CI 1.20–2.30, *p* = 0.0439). In rs1992116 (G vs. A allele), the G-allele was significantly associated with improved outcome after stroke (OR 1.67, 95% CI 1.20–2.31, *p* = 0.0385) [73]. However, the relationship between these polymorphisms and transcriptional activity is unclear and is an area in need of further research.

## 5. Matrix Metalloproteinase-3

MMP-3 (stromelysin-1), is a 51 kDa protein produced by various cells types, including fibroblasts, smooth muscle cells, and macrophages. MMP-3 degrades major ECM components—collagens I, III, IV, V, IX, and X [74], fibronectin, denatured collagens, laminin, and cartilage proteoglycans [75]. Studies have consistently noted a role for MMP-3 in progression of carotid atherosclerosis [76]. MMP-3 is upregulated in infarcted human brain after IS [57], and high *MMP-3* expression has been found to be associated with increased ischemic brain injury [77].

### Clinical Role of MMP-3 Polymorphisms

*MMP-3* polymorphisms in both promoter and coding regions have been linked to IS incidence. However, the role of *MMP-3* polymorphisms in clinical outcomes has remained controversial with conflicting associations between polymorphisms and IS incidence and clinical outcome when studied in different racial and ethnic populations [78].

A variant in the promoter region of the *MMP-3* gene -1612 (5A vs. 6A) was found to regulate the transcription of *MMP-3* [79,80]. This variant has been studied extensively with the 6A-allele found to be associated with carotid artery atherosclerosis (OR 1.58, 95% CI 1.08–2.33, *p* = 0.017) [62]. In a Finnish population, homozygotes for the 6A-allele appeared to be predisposed to arterial wall thickening [79]. Patients with the 6A/6A genotype in MMP-3 also had significantly greater intima-media thickness when compared to 5A/5A and 5A/6A genotypes (*p* < 0.03) [81]. Finally, Rundek et al. found patients with the 6A/6A genotype in MMP-3 to have significantly greater intima-media thickness in comparison to 5A/5A and 5A/6A genotypes (*p* = 0.044) [82] and therefore a potential predictor for atherosclerotic IS. The 6A-allele is associated with decreased MMP-3 expression [83].

Flex et al. found the 5A-allele of the -1171 (5A vs. 6A allele) promoter region polymorphism in *MMP-3* was significantly associated with IS incidence in an Italian population (OR 2.2, 95% CI 1.1–4.2, *p* = 0.01) [84]. The 5A-allele has higher promoter activity and higher *MMP-3* transcriptional activity [85]. However, this may not be true in a heterozygous state as no association between the -1171 5A/6A polymorphism and IS incidence was found in an Indian population [86]. Adding to the controversy, Sherva et al. studied *MMP-3* polymorphisms in 39,114 patients from the United States, Canada, Puerto Rico, and US Virgin Islands in the Genetics of Hypertension Associated Treatment study and conversely found the 6A/6A genotype to be associated with higher IS incidence in patients randomized to take lisinopril for hypertension [87].

Association of IS incidence with polymorphisms in the coding regions of MMP-3 have been shown to be dependent on ethnicity. Three SNPs [rs520540 (Ala362Ala), rs602128 (Asp96Asp), and rs679620 (Lys45Glu)] in the coding region of MMP-3 were significantly associated with IS (*p* < 0.05) in a Korean population. However, haplotype analysis revealed that no combination of these SNPs was associated with IS [78]. Conversely, Matarin et al. found no association between IS incidence and rs520540 and/or rs679620 gene polymorphisms in a North American population of European descent [88]. To date, no *MMP-3* polymorphisms have been associated with IS clinical outcome.

## 6. Matrix Metalloproteinase-9

MMP-9 (gelatinase B), is a 92 kDa protein that cleaves most ECM proteins, particularly collagen types IV [35,89] and V [90]. It also activates numerous pro-inflammatory cytokines and chemokines such as CXCL-8, interleukin 1β, and tumor necrosis factor-α. MMP-9 is involved in inflammatory responses trigged by ischemia. In addition, due to digestion of type IV collagen, it facilitates leukocytes transport across the endothelium. Moreover, by digesting occludins and claudins, MMP-9 plays an important role in BBB destruction. Finally, elevated intranuclear MMP-9 degrades poly-ADP-ribose polymerase-1 and X-ray cross-complementary factor 1 [91] and promotes accumulation of damaged DNA in neurons [92].

Thus, MMP-9 is involved in BBB destruction [93] and subsequent HT [94] that occurs particularly after thrombolysis [55,95]. MMP-9 is also involved in generation of free radicals that result in atherosclerotic lesions [96], plaque rupture in carotid atherosclerotic plaques [97], coronary artery plaque destabilization and rupture with subsequent myocardial infarctions [98,99], and embolic IS [100]. The high concentration of MMP-9 in plasma within the acute phase of IS increases the risk of HT within the ischemic core [101,102]. MMP-9 mRNA concentration was a predictor of poor outcome and mortality in IS [103].

### Clinical Role of MMP-9 Polymorphisms

Polymorphisms in the promoter and coding regions of *MMP-9* have been associated with carotid atherosclerosis. Lin et al. found that post-menopausal women carrying T-alleles (C/T and T/T) at position -1562 in the promoter region of *MMP-9* had stiffer arteries than patients with C/C genotypes even after adjusting for age and metabolic covariates [104]. Armstrong et al. evaluated the R279Q polymorphism (R vs. Q allele) and found that the R/R genotype resulted in significantly more carotid intima-media thickness when compared to the R/Q or Q/Q genotype (*p* = 0.006) [105].

Unsurprisingly, with evidence linking *MMP-9* and its polymorphisms to carotid artery atherosclerosis, studies show that *MMP-9* polymorphisms are also associated with increased IS incidence. Yuan et al. studied rs1056628 (A vs. C allele) in a Chinese population and noted dose-dependent increased expression of the C-allele (*p* < 0.01) and CC genotype (*p* < 0.05) in IS patients when compared to controls [106]. The promoter region -1562 (C vs. T allele) polymorphism was also shown to influence IS incidence with the T-allele associated with increased IS incidence (OR 1.543, 95% CI 1.144–2.080, *p* = 0.004) [72].

Although extensive evidence shows an association between *MMP-9* expression and HT [94,102] particularly after administration of intravenous thrombolysis [55,95,107], the effect of *MMP-9* polymorphisms on HT susceptibility is unclear. Zhang et al. evaluated the -1562 (C vs. T allele) promoter region polymorphism in a Chinese population and found that the C/C genotype was associated with significantly more HT compared to the C/T and T/T genotype (*p* = 0.037), and the frequency of the C-allele was significantly higher in patients with HT compared to the T-allele (*p* = 0.035) [108]. However, the -1562 C/T polymorphism did not play a role in HT after systemic thrombolysis in a Mediterranean population [109].

Despite extensive evidence linking *MMP-9* expression with worse clinical outcome in IS, the role of *MMP-9* polymorphisms remains unclear [110] and to date, no *MMP-9* polymorphisms have been associated with clinical outcome in IS [73].

## 7. Matrix Metalloproteinase-12

Macrophage metalloelastase (MMP-12) is secreted as a 54 kDa pro-form protein that undergoes self-activation through autolytic processing to produce 45 kDa and 22 kDa active forms. MMP-12 plays a significant role in the pathophysiology of IS. It displays a broad substrate specificity, including degradation of the extracellular proteins—fibronectin, laminin, elastin, vitronectin, type IV collagen, and heparan sulfate [111,112,113]. Thus, MMP-12 enables macrophages to penetrate injured tissues during inflammation and facilitates monocytes transmigration across the endothelium [111,112]. In addition to digesting basement membrane components, MMP-12 also activates MMP-2 and MMP-3 [114], thus, synergistically activating a cascade of proteolytic processes, which ultimately lead to BBB disruption.

### Clinical Role of MMP-12 Polymorphisms

Similar to other MMPs, *MMP-12* polymorphisms have been found to play a role in the development of carotid atherosclerosis, which may be a potential mechanism in the association between certain alleles and IS incidence. The *MMP-12* polymorphism rs660599 was found to be significantly overexpressed in patients with carotid plaques compared to atherosclerosis-free controls (*p* = 1.2 × 10^−15^) [115].

Chehaibi et al. suggested that the *MMP-12* promoter polymorphism rs2276109 (A vs. G allele) may be a risk factor for IS in diabetic patients. They reported that the *MMP-12* rs2276109 AA genotype was significantly associated with IS incidence in a Tunisian population (OR 1.79, 95% CI 1.29–2.69, *p* = 0.001). This relationship, however, was not found to be replicated in a non-diabetic population. The A-allele of the *MMP-12* rs2276109 polymorphism (A vs. G allele) has been shown to have a higher affinity for the transcription factor activator protein-1 [116], resulting in increased expression of MMP-12 [61].

Similarly, the *MMP-12* rs652438 (357 Asn/Ser) polymorphism, located in the coding region of the hemopexin domain that is responsible for MMP-12 activity [117], was found to be associated with IS incidence in diabetic individuals (adjusted OR 1.68, 95% CI 1.22–2.56, *p* = 0.001) [61]. However, to date, no direct association between *MMP-12* polymorphisms and clinical outcome in IS has been reported.

## 8. Conclusions

The clinical roles for *MMP* polymorphisms are complex due to multifactorial mechanisms. Progression of carotid artery atherosclerosis (Table 1) represents a major pathophysiological mechanism responsible for IS incidence. However, as shown when comparing *MMP* polymorphisms responsible for IS incidence (Table 2) with polymorphisms responsible for carotid artery atherosclerosis, these polymorphisms do not necessarily lead to straightforward associations. Similarly, the multifactorial mechanisms and past medical history associated with clinical outcome after IS makes targeting the association between specific polymorphisms and clinical outcome extremely difficult (Table 3). Although several MMPs have a well-established relationship with HT, there is a less clear relationship between certain *MMP* polymorphisms and HT (Table 4).

## 9. Future Studies of Investigation

The classification of MMPs into their respective roles represents a challenging question for future study. First, individual MMPs must be studied further to evaluate for their respective roles in CVD pathology. The most important and widely-studied MMPs appear to be MMP-2, 3, 9, and 12. Second, once the important MMPs are targeted, the functional genetic variations of each of these MMPs must be addressed. Third, the role of these *MMP* polymorphisms in protein activity and transcriptional activity must be better characterized. Fourth, the association between these polymorphisms and ethnic disparities in clinical outcome after stroke must be related [8]. And finally, as ubiquitous proteins that appear to have both pathological and neuroprotective roles [118], the mechanism of action of these MMPs must be better characterized.

## Figures and Tables

**Table 1:** Matrix metalloproteinase (MMP) polymorphism relation to carotid artery atherosclerosis and intima-media thickness.

MMP Studied	Polymorphism Studied	Gene Region	Population and/or Location	Findings	Transcriptional Activity	Reference
MMP-1	-1607 (1G/2G)	Promoter	Tunisian	No difference	Increased with 2G-allele	[62]
MMP-3	5A/6A	Promoter	Tunisian	6A-allele associated with ICA atherosclerosis *	Decreased with 6A-allele	[62]
MMP-3	5A/6A	Promoter	Unknown	6A-allele associated with ICA atherosclerosis ±	Decreased with 6A-allele	[81]
MMP-3	-1612 (5A/ 6A)	Promoter	Hispanic, Black, and White	6A/6A genotype associated with greater carotid atherosclerosis ±	Decreased with 6A-allele	[82]
MMP-9	-1562 (T/C)	Promoter	Chinese, post-menopausal women	T-allele associated with stiffer arteries ±	Increased with C-allele	[104]
MMP-9	R279Q (R/Q)	Coding	German	R/R genotype associated with greater mean carotid intima-media thickness *	n/a	[105]
MMP-12	-rs660599	Coding	Unknown	SNP overexpressed in patients with carotid plaques *	n/a	[115]

* Association found via logistic regression analysis; ± Association found via multivariable linear regression analysis; n/a = not applicable.

**Table 2:** MMP polymorphism relation to ischemic stroke incidence.

MMP Studied	Polymorphism Studied	Gene Region	Population and/or Location	Finding	Transcriptional Activity	Reference
MMP-1	-1607 (1G/2G)	Promoter	Tunisian	No difference	Increased with 2G-allele	[61]
MMP-2	-1306 (C/T)	Promoter	Chinese	C-allele associated with leukoaraiosis *	Increased with C-allele	[71]
MMP-2	-1306 (C/T)	Promoter	Chinese	No difference	Increased with C-allele	[72]
MMP-2	-735 (C/T)	Promoter	Chinese	C-allele associated with greater incidence of IS *	Increased with C-allele	[72]
MMP-3	-1171 (5A/6A)	Promoter	Italian	5A-allele associated with greater incidence of IS *	Increased with 5A-allele	[84]
MMP-3	-5A/6A	Promoter	Multi-racial	6A/6A genotype associated with greater incidence of IS in patients taking lisinopril	Increased with 5A-allele	[87]
MMP-3	-rs520540 (G/A)	Coding	Korean	G-allele associated with greater incidence of IS	n/a	[78]
MMP-3	-rs520540 (G/A)	Coding	Unknown	No difference	n/a	[88]
MMP-3	-rs3025058 (5A/6A)	Coding	Korean	G/G and G/A genotype associated with greater incidence of IS	n/a	[78]
MMP-3	-rs3025058 (5A/6A)	Coding	Unknown	No difference	n/a	[88]
MMP-3	-rs679620 (G/A)	Coding	Korean	G-allele associated with greater incidence of IS in women only	n/a	[78]
MMP-3	-rs679620 (G/A)	Coding	Unknown	No difference	n/a	[88]
MMP-3	-rs602128 (C/T)	Coding	Korean	C-allele associated with greater incidence of IS	n/a	[78]
MMP-9	-rs1056628 (A/C)	Coding	Chinese	C/C genotype associated with greater incidence of IS	n/a	[106]
MMP-9	-1562 (C/T)	Promoter	Chinese	T/T genotype associated with greater incidence of IS *	Increased with T-allele	[72]
MMP-12	-rs2276109 [82] (A/G)	Promoter	Tunisian	A/A genotype associated with greater incidence of IS *	Increased with A-allele	[61]
MMP-12	-rs652438 [1082] (A/G)	Promoter	Tunisian	A/A genotype associated with greater incidence of IS *	Increased with A-allele	[61]
MMP-12	-rs660599	Coding	Unknown	Replication of SNP associated with greater incidence of IS with large-artery mechanism *	n/a	[115]

IS = ischemic stroke. SNP = single nucleotide polymorphism; * Association found via logistic regression analysis; n/a = not applicable.

**Table 3:** MMP polymorphism relation to clinical outcome.

MMP Studied	Polymorphism Studied	Gene Region	Population and/or Location	Finding	Transcriptional Activity	Reference
MMP-2	-rs2241145 (G/C)	Intronic	Portuguese	G-allele associated with improved clinical outcome *	n/a	[73]
MMP-2	-rs1992116 (G/A)	Intronic	Portuguese	G-allele associated with improved clinical outcome *	n/a	[73]

* Association found via logistic regression analysis; n/a = not applicable.

**Table 4:** MMP Polymorphism relation to hemorrhagic transformation.

MMP Studied	Polymorphism Studied	Gene Region	Population and/or Location	Finding	Transcriptional Activity	Reference
MMP-9	-1562 C/T	Promoter	Chinese	C-allele associated with higher rate of HT	Increased with C-allele	[108]
MMP-9	-1562 C/T	Promoter	Mediterranean	No difference	Increased with C-allele	[109]

HT = hemorrhagic transformation.
